# Alignment of behaviour and tDCS stimulation site induces maximum response: evidence from online tDCS and ERP

**DOI:** 10.1038/s41598-024-68691-2

**Published:** 2024-08-24

**Authors:** Sagarika Bhattacharjee, Rajan Kashyap, Kaviraja Udupa, Shahid Bashir, Ganesan Venkatsubramanian, Kenichi Oishi, John E. Desmond, Brenda Rapp, S. H. Annabel Chen

**Affiliations:** 1 Department of Neurophysiology, National Institute of Mental Health And Neuro Sciences (NIMHANS), Hosur Road, Bangalore, Karnataka 560029 India; 2 Psychology, School of Social Sciences, Nanyang Technological University, Singapore, Singapore; 3 Department of Neuroimaging and Interventional Radiology, National Institute of Mental Health And Neuro Sciences (NIMHANS), Hosur Road, Bangalore, Karnataka 560029 India; 4 Neuroscience Center, King Fahad Specialist Hospital Dammam, Dammam, Saudi Arabia; 5 Department of Psychiatry, National Institute of Mental Health And Neuro Sciences (NIMHANS), Bengaluru, India; 6 The Johns Hopkins University, School of Medicine, Baltimore, USA; 7 Department of Cognitive Science, The Johns Hopkins University, Baltimore, USA; 8 Centre for Research in Child Development (CRCD), National Institute of Education, Singapore, Singapore; 9 Lee Kong Chian School of Medicine (LKC Medicine), Nanyang Technological University, Singapore, Singapore

**Keywords:** tDCS, Word priming, Event-related potential (ERP), Cognitive neuroscience, Learning and memory

## Abstract

tDCS modulates the activity of the neuronal networks to induce the desired behavioural changes. Two factors determine its effectiveness- (1) whether the network being stimulated is relevant to the task, and (2) if there is a scope for improvement in behavioral performance. To explore this, both dorsal (sub-lexical) and ventral (lexical) reading networks were stimulated (20 min, 2 mA) in 25 healthy young volunteers. Participants performed two reading tasks with different levels of lexical involvement: word fragment completion tasks (WCT) and word association tasks (WAT), while event-related potentials (ERPs) were recorded simultaneously. The study used a within-subject design over three sessions, comparing various electrode montages targeting the dorsal pathway's left inferior parietal lobule or the ventral reading pathway's left middle temporal lobule, as well as sham stimulation. The impact of tDCS sessions (dorsal, ventral, & sham) and task type (WCT & WAT) on priming effects (primed vs. unprimed) of behavioral performance (accuracy and reaction times), and ERP parameters (N400 amplitudes and latencies) were statistically analyzed.It was found that tDCS modulated the performance of WAT only (a task with a lower priming effect). The failure to modulate WCT (larger priming effect) indicated that tDCS was effective for conditions with room for improvement compared to a task where performance has reached the ceiling. Ventral stimulation enhanced accuracy in the WAT condition and shortened the N400 latency of the priming effect. In contrast, dorsal stimulation delayed the priming effect reaction time in the WAT condition and enhanced the N400 amplitude. To conclude, enhancement in performance due to tDCS occurs when the network (ventral) being stimulated aligns with the cognitive demands of the task and there is a scope for improvement.

## Introduction

Transcranial direct current stimulation (tDCS) can modulate the activity of the underlying brain networks involved in the performance of the task. The tasks that are processed by multiple pathways in the brain, the specificity in stimulating the underlying network is vital in shaping the induced neuronal plasticity^1,2^. To explore the specificity of tDCS effects, selective stimulation of the networks involved in processing the task becomes important. Reading is one such behaviour that is supported by two underlying networks whose degree of utilization can be independently manipulated. Reading involves two major cognitive processes -lexical and sub-lexical- supported by distinct neural networks. Lexical processing (identifying the meaning of a written word) is performed by a ventral pathway comprised of the left middle temporal gyrus, basal temporal area, and inferior frontal gyrus (pars orbitalis and pars triangularis). In contrast, sub-lexically processed orthographic-phonology conversion translates letters to sounds employing a dorsal pathway that consists of regions in the inferior parietal lobule, superior temporal gyrus, and inferior frontal gyrus (pars opercularis  ^2–4^). These two networks lie close within the cerebral cortex.

In our previous work^5–8^, we used simulation (Systematic Approach for tDCS Analysis (SATA) toolbox) to derive two appropriate montages that could selectively stimulate the left inferior parietal lobule of the dorsal network and the ventral network's left middle/inferior temporal gyrus. Dorsal montage consisting of the anode at CP5 and cathode at CZ and ventral montage fitting the anode at TP7 and cathode at the nape of the neck were shown to have the least amount of overlap in their spread of current. In an experimental study, we used these montages to investigate the effect of selective dorsal and ventral reading network stimulation on English-Chinese bilinguals^1,9^. We found that tDCS applied to the dorsal network enhanced English reading capability, but the ventral network stimulation failed to improve performance (compared to sham stimulation). On the other hand, both the dorsal and ventral network stimulation enhanced the reading of the Chinese language. It was postulated that the bilinguals, who were highly proficient in English (as shown by their behaviour proficiency scores), mainly relied on the ventral network to process the language, leaving no room for improvement following tDCS. In contrast, the less proficient bilinguals in Chinese had a broader scope for improvement in response to both dorsal and ventral network stimulation. It was also found that lower the script-specific sub-lexical proficiency scores, the higher the effectiveness of dorsal network tDCS.

Given these findings, it can be hypothesized that (1) tDCS is more effective in tasks where the ceiling point in performance has not been reached, and there is room for improvement, and (2) maximal tDCS benefit is achieved following stimulation of the specific network (dorsal/ventral) involved in processing a corresponding task (e.g., word reading).This could be investigated experimentally by stimulating dorsal (sub-lexical) and ventral (lexical) networks while the tasks with varying lexical demands are performed. The prediction would be that, although both pathways are involved in reading, because of the high lexical demands of the tasks, performance will be enhanced following tDCS to the ventral pathway (compared to the dorsal pathway). Reading tasks that involve priming of the words can be used to assess the performance as the degree of priming effect can be modulated by manipulating the task characteristics^10^. To further understand the neurophysiological basis of the phenomenon, tDCS can be combined with an electroencephalogram (EEG) and task specific modulation in the waveforms can be obtained.

The present study compares two-word priming tasks to address these questions: word fragment completion tasks (WCT) and word association tasks (WAT) in three sessions of dorsal, ventral, and sham stimulation conducted in a within-subject design. The WCT requires participants to complete a stimulus consisting of word fragments (e.g. test: B_H_ _ D) in primed and unprimed conditions. In the primed condition, a complete word (e.g., BEHIND) is presented in the study phase (primed condition), while in the unprimed condition, it was not. The reader responds with a complete word while the experimenter records the accuracy and the time required to respond (reaction time) to repeated stimuli. Similarly, WAT requires responding to a stimulus word with a semantically associated word (e.g. test: BEACH) under both primed and unprimed conditions. Some trials are primed with a previously presented semantically related word (e.g. SAND) in the study phase, while in the unprimed condition, no related word was presented. Benefits for the primed vs unprimed conditions typically involve increased accuracy and faster responses. These behavioural benefits are accompanied by decreased amplitude and latencies of specific ERP components (e.g. N400) for repeated (primed) stimuli^11^. Amongst these tasks, the priming effect (primed minus unprimed) in the WCT is expected to reach the ceiling point in healthy individuals, whereas the priming effect seen in the WAT will have room for improvement. This is because priming effects increase with enhanced prime-target relatedness (which is more in the case of WCT)^10^, and the neural correlates of these effects will manifest in the ERPs. Thus, two main findings are expected from the present experiment highlighting the importance of (1) *tDCS specificity*- tDCS targeting the ventral pathway will be more effective than dorsal pathway stimulation; and (2) *Room for improvement*- the desired tDCS effect will be pronounced for the more challenging WAT compared to WCT.

## Methodology

### Participant description

Forty-five young adults (aged 21–35) were recruited through advertisements to participate in the study. They were asked to complete an online questionnaire that self-rated their English proficiency level (on a scale of 1 to 10). Participants who rated their proficiency above eight were recruited for a behavioural session that measured their English proficiency. Out of the 45, only 25 participants, with > 8 self-rated and > 75% measured proficiency scores, were recruited for the study. None of the participants had reading difficulties, neurological or psychiatric illness, history of head trauma, personal or family history of epilepsy, or any prescription of psychiatric medications. Pregnant and breastfeeding women, and participants with scalp abrasions or skin diseases were also excluded. All study protocols were preregistered in the open science forum^12^ and approved for ethics by the Institutional Review Board at Nanyang Technological University with no: IRB-2017-09-001-06. All experiments were performed in accordance with relevant guidelines and regulations with participants' written informed consent.

### Study materials

#### Description of the word completion and word association tasks

**Figure 1:**
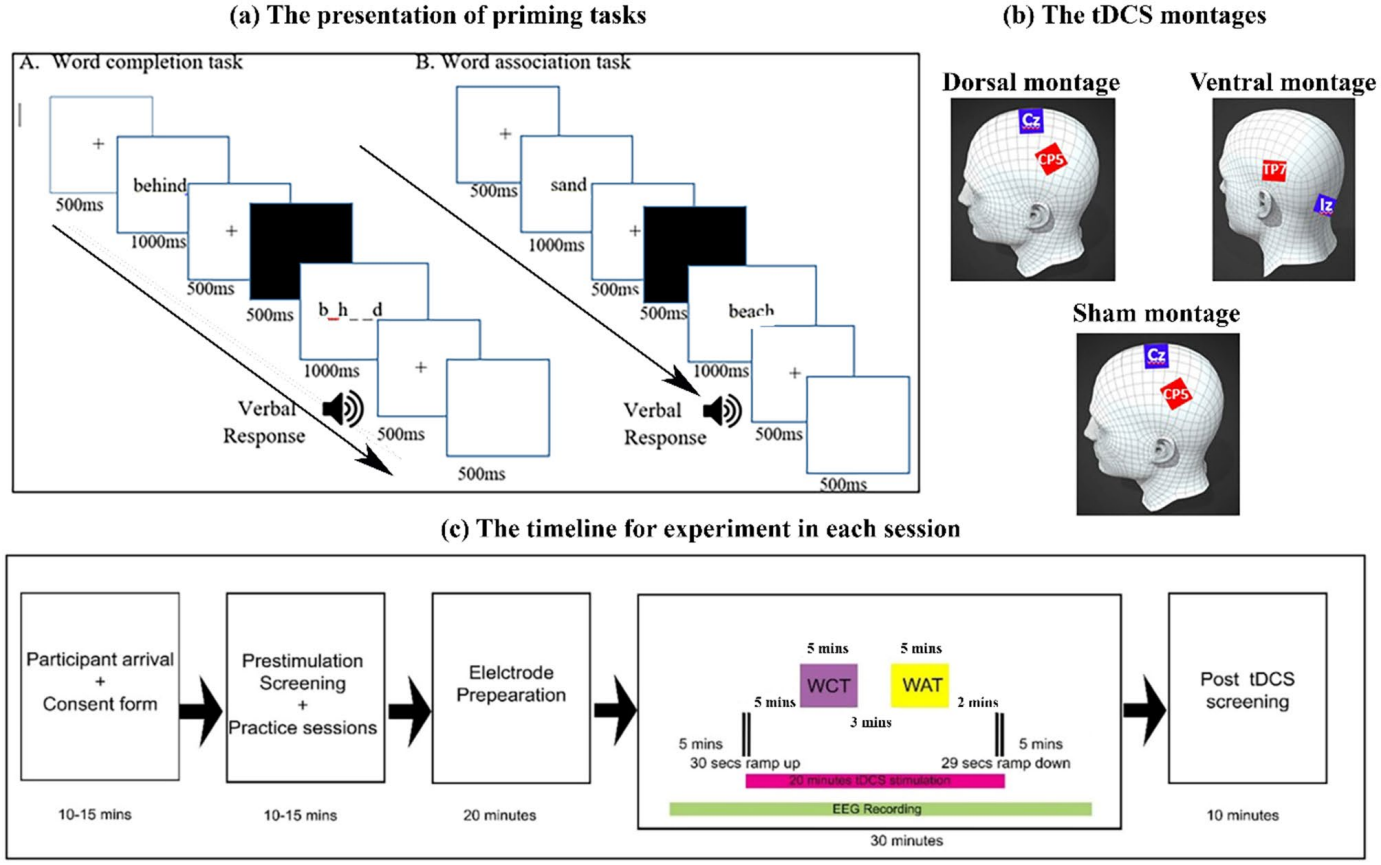
(**a**) The trial structures for the Word Completion (WCB) and Word Association (WAT) Tasks. (**b**) The dorsal and ventral network, and sham montages used in the experiment (**c**) The timeline of events for each of the three sessions: dorsal active tDCS, ventral active tDCS, and sham.

Two priming tasks, WCT and WAT, were administered using an event-related design appropriate for collecting ERPs. Tasks were presented using Eprime software^13^. For both tasks, each trial consisted of an initial fixation cross of 500 ms followed by a presentation of a "priming phase" for 1000 ms. Then, a fixation cross and blank screen appeared for 500 ms each, followed by the "test phase" for 1000 ms, as shown in Fig. 1(a). In WCT, the prime phase contained a complete word (e.g., BEHIND) in the primed condition, whereas it was left blank in the unprimed condition. In the test phase, the stimulus consisted of an incomplete word (e.g., B_H_ _ D) in primed and unprimed conditions. Participants were instructed to complete the incomplete word with the first word that came to mind. They were told that using a previously seen word was acceptable and to leave it blank if nothing came to mind. The oral responses were recorded with a voice key attached to the Chronos device of Eprime^14^, which records both the voice and RTs of the response. As in the WCT, the priming phase in WAT consisted of a complete word (e.g., SAND) or a blank in the primed and unprimed conditions, respectively. Participants were instructed to respond as quickly as possible with the first word that came to mind related in meaning if the test word (e.g., BEACH) that they had just seen. They were told that using a previously seen word was acceptable and to leave a blank if nothing came to mind.

#### Preparation of the priming tasks for Singaporeans

Stimuli for WCT and WAT were obtained from Anderson et al.^15,16^ and Fitzpatrick et al.^17^ respectively. All the fragments in WCT had unique solutions. Since these tasks had been generated and validated in the United Kingdom, the most probable response for each stimulus was again estimated at the experimental site for 33 young (16 to 25) Singaporean individuals. They were asked to respond to the test phase stimuli of these two tasks, either word fragments (WCT) or complete words (WAT), with the first word that came to mind. Out of 128 words in the original WCT task and 102 words in the original WAT task, 93 stimuli were selected for each task for this study based on the following criteria:

1. If the response with the highest frequency amongst the Singaporean population matched the target response in the original task, the stimulus was retained.
2. If the response with the highest frequency in the Singaporean population differed from the response in the original task, the stimulus was included, but the response in the original task was replaced with the response with the highest frequency in the Singaporean population.
3. The stimulus was discarded if two responses were "tied" for the highest frequency in the Singaporean population.

The responses thus identified for each stimulus were regarded as the baseline responses, which corresponded to the expected response for Singaporean participants in an unprimed condition and were used as primes for primed conditions. For each task, three lists (103 items each) were created, one each for ventral stimulation, dorsal stimulation and sham. The lists were created by shuffling the words in primed (52 stimuli) and unprimed conditions (51 stimuli) in such a way that the primed and unprimed conditions of the final three lists did not vary statistically on length, frequency, number of syllables, number of phonemes, bigram frequency, orthographic neighbourhood, and phonological neighbourhood, naming reaction time obtained from corpus od English Lexicon Project^18^. The trial sequence for the two tasks is shown in Figure 1(a).

#### Validation of the priming tasks conducted before the tDCS experiment

Thirty-three healthy individuals were recruited to perform the WAT and WCT tasks in a within-person design to validate previously reported response patterns with the stimulus lists and population (Singaporean) used in this study. Participants were administered both tasks in a single session. The three versions of each task (WCT and WAT) were randomized and counterbalanced across participants, and accuracies (ACCs) and reaction times (RTs) were recorded. In both tasks, in the primed and unprimed conditions, if participants responded to the stimulus in the "test phase" with the prime word, it was marked as "correct." The "priming effect" corresponded to the difference between the ACCs and RTs of "correct" responses in the primed compared to the unprimed conditions. We expect to replicate the previously reported findings of priming effects in WCT and WAT, where the priming effect of WCT would be larger than the WAT (Henson et al.^19^). To validate this, we performed a logistic-linear mixed effect model (LMEM) analysis with ACCs (LMEM 1a, see Table 1) and regular LMEM with RTs (LMEM 1b, see Table 1) as the dependent variables and task-type (WCT and WAT), task-version (Lists 1, 2, and 3) and primed/un-primed condition (P and U) as fixed factors. The 25 Subjects and 103 items for each list were treated as crossed random factors to demonstrate the variations among items across all individuals, as well as the differences within individuals. The main effect of the Task-Version was expected to be non-significant in LMEMs 1a and b. Thus, to avoid the three-way interaction between the three fixed factors in LMEM1 (task-type, task-list, and priming-condition), we planned to perform another LMEM analysis for ACCs and RTs (LMEM 2a and 2b, see Table 1), with task-type and priming-condition as fixed factors and subjects and items as crossed random factors.

**Table 1:** Summarises the LMEM, equations, significant expected and unexpected findings and conclusions drawn.

LMEMs	Equation	Significant findings	Conclusions
*LMEMs of behavioural effects for tasks administered before the tDCS experiment:* To investigate whether the three tasks list to be used in the ventral, dorsal and sham sessions are equivalent and whether the priming effect of WAT > WCT			
LMEM 1a and b	$$\begin{aligned}1\text{a}.\text{ ACC }\sim \text{ task}\_\text{type}& *\text{ P}\_\text{U }&\\ *\text{ Var}\_\text{task }&\\+ (1|\text{Subject})&\\+ (1\text{item}) \end{aligned}$$ $$\begin{aligned}1\text{b}.\text{ RTs }\sim \text{ task}\_\text{type}*\text{ P}\_\text{U }\\ *\text{ Var}\_\text{task }&\\ +(1|\text{Subject}) \\+ (1|\text{item})\end{aligned}$$	•The three versions of the stimulus list do not have significant main or interaction effects	•The three versions of the stimulus lists are equivalent
LMEM 2a and b	$$\begin{aligned}2\text{a}.\text{ ACC }\sim \text{ task}\_\text{type }*\text{ P}\_\text{U }+ (|\text{Subject}) + (1|\text{item})\end{aligned}$$ $$\begin{aligned} 2\text{b}.\text{ RTs }\sim \text{ task}\_\text{type } *\text{ P}\_\text{U }\\+ (1|\text{Subject}) \\+ (1|\text{item})\end{aligned}$$	•In terms of accuracies, the priming effect for WCT is larger than for WAT •For both tasks, responses were faster for WCT than WAT, and within each task, responses were faster for the primed condition than the unprimed condition	•WCT accuracies are at the ceiling point •There is room for improvement in the accuracy of the WAT
*Effect of tDCS on task performance:* To investigate the effect of tDCS on task performance			
LMEM 3 and 4	$$\begin{aligned}3.\text{ ACC }\sim \text{ task}\_\text{type}*\text{ P}\_\text{U}*\text{ tDCS}\\ + (1|\text{Subject})\\ + (1\text{item})\end{aligned}$$ $$\begin{aligned}4.\text{ RTs }\sim \text{ task}\_\text{type }*\text{ P}\_\text{U }*\text{ tDCS } \\ + (1|\text{Subject}) \\ + (1|\text{item})\end{aligned}$$	•Accuracies in WAT are enhanced following ventral stimulation •RTs in WAT slowed down following dorsal stimulation •Stimulation-sessions do not affect behaviours in WCT	•tDCS are effective for difficult WAT tasks where there is room for improvement •Stimulation of the ventral pathway (lexical) is beneficial •The slowing of the RT in WAT in response to dorsal stimulation was unexpected
Effect of tDCS on priming effect (primed-unprimed) on N400 component			
LMEM 5	$5.\text{Latency}\_$PE^Relevant^ $\sim \text{ task}\_\text{type}*\text{ tDCS}+ (1|\text{Subject})$	The latency of the priming effect in the WAT task was significantly reduced following ventral stimulation compared to sham	Ventral stimulation accelerates faster brain response for the WAT, thereby enhancing the accuracy
LMEM 6	$6.\text{ Amplitude}$ _PE^Relevant^ $\sim \text{ task}\_\text{type}*\text{ tDCS}+ (1|\text{Subject})$	The amplitude of WAT priming effects was significantly enhanced following dorsal stimulation compared to sham	Dorsal stimulation causes greater activation of neuronal resources for WAT, thereby slowing the reaction times

*task_type(WAT or WCT), Var_task (List1, 2, and 3), P_U(Primed\Unprimed), tDCS(dorsal\ventral\sham), ACCs = accuracy, RT = Reaction time, PE^relevant^ = Relevant Priming effect for task.

### tDCS experiment

The experiment consisted of tDCS stimulation while performing two reading tasks (WCT and WAT) with simultaneous EEG recording. Participants were asked to undertake a practice trial version of the behavioural WCT and WAT tasks with stimuli that were different from the experimental stimuli (for details, refer to “Description of the word completion and word association tasks”) to ensure they understood the instructions correctly.

Participants were asked to attend three sessions, each involving one type of stimulation (dorsal, ventral, or sham). The stimulation type was double-blinded for both the participant and the experimenter, using a preassigned numerical code to initiate stimulation via the Neuroconn device^20^. Before placing the stimulation electrodes, the participant's scalp was inspected for cuts, abrasions, or skin conditions. Conductivity was improved by removing/adjusting the hairs from the stimulation and recording sites, securing them with plastic clips, and cleaning the scalp with alcohol swabs (to remove dirt and excess oil). The scalp was then allowed to dry. The vertex or Cz location was determined by measuring the distance from the nasion to inion and marking the midpoint with a skin-friendly marker.

tDCS electrodes were placed on the appropriate montage markings using Ten20 thick conductive paste to ensure they stayed in place. The montages were chosen based on previous studies to optimize the selective stimulation of the dorsal and ventral reading pathways^1,5^. For stimulation, 3 × 3 cm^2^ electrodes were used with the anode at CP5 and cathode at Cz for the dorsal network, and anode at TP7 and cathode at the nape of the neck for the ventral network (see Fig. 1(b) for montage configurations). The dorsal pathway montage was also used for sham stimulation, as its configuration was not expected to influence the outcome.

After confirming the tDCS electrode conductivity with the Neuroconn device, an EEG cap with 64 channels from the Deymed EEG system was placed over them, ensuring that the vertex aligned with Cz on the cap. Electrolyte gel was applied to the EEG electrodes using a curved syringe, taking care to prevent contact between electrodes (via the gel) to avoid superficial shunting of current. Reference electrodes were attached to one of the mastoids without touching each other. Two additional EEG electrodes with skin-friendly adhesive tape was placed above and below the eyes to record eye movements. Once preparations were complete, electrode impedance was checked and maintained below 10-15KΩ for tDCS and 5KΩs for EEG. EEG recording began 5 min before the start of tDCS stimulation and continued until 5 min post-stimulation. Active tDCS stimulation lasted for 20 min. The current was gradually ramped up for 30 s and maintained for the rest of the duration at an intensity of 2 mA before it ramped down in the last 30 s. In the sham condition, the current was ramped up and ramped down during the initial 30 s, and participants received no further stimulation for the experiment.

Participants were asked to perform the two tasks, WCT and WAT (each with a list of 93 stimuli for 5 min), starting 2.5 min following the start of stimulation, with a 1-min gap between tasks. Three types of randomizations and counterbalancing were carried out across the three sessions and participants with randomization of (1) stimulation-session (dorsal, ventral, and sham), (2) task-type (WCT and WAT), and 3) task-version (1, 2 and 3). After the stimulation was completed, the participants were asked to rate the tolerability of the procedure on a scale of 1 (mild) to 5 (severe) for common side effects (i.e., headache, neck pain, scalp pain, tingling, itching, burning sensation, skin redness, sleepiness, trouble concentrating, acute mood changes, and others). The success of blinding was also evaluated by asking the participants to guess the stimulation session as active/"real," sham/"fake," or "I don't know." A timeline of the entire procedure is depicted in Fig. 1(c).

### Electroencephalography (EEG) recording

64-channel EEG equipment (Daymed Truescan, Germany) was used to record the EEG signals using Ag/AgCl electrodes mounted on an elastic cap. The ground electrode was placed in the forehead position. Two reference electrodes were placed in both the left and right mastoid. Bipolar electrodes were placed at the infraorbital ridge and the outer canthus of the right and left eyes to record the horizontal and vertical eye movements. The electrode impedance was kept below 5 kOhm. The electrophysiological signals were filtered with a bandpass of 0.1–1000 Hz (half-amplitude cut-offs) and digitized at a rate of 5000 Hz using a 16-bit A/D converter. The EEG recording started 5 min before stimulation and continued 5 min post-stimulation, providing the necessary time for stabilizing the EEG signal.

### Statistical analysis

#### Behavioural analysis of the priming tasks conducted during tDCS

Separate logistic—LMEM evaluated the effect of tDCS on ACCs (LMEM 3, see Table 1) and the regular LMEM on RTs (LMEM 4, see Table 1) of both tasks. In both models, task-type (WCT and WAT), stimulation session (dorsal, ventral, and sham), and priming condition (Primed/Unprimed) were included as fixed factors. Twenty-five subjects and 103 items for each list were treated as crossed random factors. Appropriate contrast was set for stimulation conditions: dorsal versus sham as [1, 0, − 1]; and ventral versus sham as [0, 1, − 1]. Similarly, primed was contrasted against unprimed, and WCT was contrasted against WAT as [1, − 1]. The p-values were calculated using the likelihood ratio test, and estimated marginal means were determined as least square means. The values were then tested for significance using Bonferroni's correction.

#### EEG pre-processing

All EEG analyses were conducted using the EEGLAB (version 9.046) Toolbox^21^ and MATLAB 2019a. Following pre-processing pipeline was followed in line with the previous studies that used a combination of tDCS and EEG^22^.

1. *Filtering and interpolation of bad channels:* A bandpass filter (0.5–30 Hz) was applied to the signals to remove low-frequency drifts and high-frequency noise. Electrodes affected by current due to tDCS that appeared as straight lines in the raw data were removed by visual inspection as bad channels. The signals in the missing electrode were then interpolated using a linear combination of the potentials of the four nearest electrodes. tDCS-affected electrodes were identified only in the active tDCS sessions for a few participants (one electrode for 9 participants and two electrodes for 2 participants) and mostly surrounding the tDCS electrodes (i.e., CP5, CZ, TP7, and Iz). The interpolated electrode was not considered in the statistical analysis and was only used for the graphical display of the topographic images.
2. *Inspection and rejection of data visually*: Visual inspection rejected bad portions of the continuous data. These were mainly the portions present during the ramp-up and -down of the tDCS current, as it takes some time for the EEG signal to adjust with the influx of current.
3. *Decomposing the data by ICA and artefact rejection:* The data was decomposed using independent component analysis (ICA), especially selecting the default “runica” algorithm in the EEGLAB. EEGLAB extension of SASICA^23^, ADJUST^24^, and FASTER^25^ provided objective measures like correlation with vertical and horizontal electrooculography electrodes, low autocorrelation of time-course, focal channel topography, focal trial activity, residual variance, correlation with bad channels, spatial and temporal kurtosis, slope of the power spectrum, hurst exponent, median slope of time-course, spatial average and variance difference and maximum epoch variance for each of 64 ICA components (for details, refer to Chaumon et al.^23^). These parameters guide the identification of potential artifact components such as blinks, saccades, muscle noise, bad channels, rare events, heart components, and sudden shifts in amplitude. To support the automated procedure and avoid mistakenly removing neural signals, we adhered to clear guidelines regarding the expected properties of each potential artifact and neural signal, as established by Chaumen et al.^28^. Manual supervision was employed to ensure accuracy in the decision-making process of rejecting artifacts.
4. *Epoching and baseline correction*: The data were segmented into epochs (-200 to 1995 ms) for each task-type (WCT/WAT) and priming-condition (Primed/Unprimed). Each epoch was baseline-corrected for 200 ms by subtracting the mean amplitude of the pre-stimulus period from the entire epoch. Trials with significant drift were manually removed.
5. *Averaging and ERP Component Analysis*: Epochs were averaged for each condition (dorsal, ventral, and sham) and task-type (WCT, WAT) to obtain the event-related potentials (ERPs). The N400 ERP component was identified and analyzed based on its latency.

#### Priming effect analysis

The ERPs were generated by sub-epoching the data from −100 to 800 ms and averaging the trials for each channel in each participant. Grand average waves were calculated by combining the ERP waves of each person for each of the four experimental conditions: WAT_P, WAT_U, WCT_P, and WCT_U. The priming effect for each subject was calculated by subtracting the unprimed condition from the primed condition, i.e.,

$$Priming\, effect \left(PE\right)word\, association\, task (P{E}_{WAT})=WAT\_P-WAT\_U$$

$$Priming\, effect \left(PE\right)word\, completion\, task\left(P{E}_{WCT}\right)=WCT\_P-WCT\_U.$$

The priming effect (PE^WAT^ and PE^WCT^) of the channels was reduced (dimensionality reduction) using the principal component analysis, and up to 6 components were derived. The first component was extracted for further analyses, accounting for ~ 93% of the variance across all subjects (93.4 ± 3.4). Since studies have reported the priming effect for word reading task to be significant ~ 400 ms (i.e., N400)^26^, we epoched the signal of the first component from 200 to 500 ms (encompassing the N400 envelope)^27^. This relevant envelope of the priming effect for WAT and WCT will be referred to as PE^Relevant_WAT^ and PE^Relevant_WCT^, respectively.

#### Statistical analyses of ERPs

LMEM 5 and 6 (see Table 1) evaluated the effect of tDCS on the priming effects (primed−unprimed) to understand the neurophysiological basis of observed behavioural changes determined by LMEM 3 and 4. Thus, the latency (LMEM5) and amplitude (LMEM6) of priming effects were modelled for tDCS stimulation (dorsal, ventral and sham), and task-type (WAT and WCT) as fixed factors. Unlike LMEM 3 and 4, only 25 subjects were treated as crossed random factors to account for interindividual differences in the priming effect. Item-wise differences within an individual was not considered because the priming effect was derived from the grand averaged ERP data of all trials for each individual. Appropriate contrasts were set for stimulation conditions: dorsal versus sham as [1, 0, -1]; and ventral versus sham as [0, 1, -1]. Similarly, WCT was contrasted against WAT as [1, -1]. The p-values were calculated using the likelihood ratio test, and estimated marginal means were determined as least square means. They were then corrected for multiple comparison using Bonferroni's correction method.

## Results

Twenty-five right-handed English bilingual speakers (12 females, mean age = 22.10 ± 3.9 SD) were qualified for this study after proficiency testing. All participants tolerated the tDCS protocol. The tingling was the most commonly reported side effect (94.65% dorsal active; 100% ventral active; 91% sham), followed by mild itching, burning sensation, and sleepiness. Blinding was adequate, as no significant group differences were found for the participants stating about their Stimulation session (χ^2^ = 0.829 p = 0.301).

### Behavioural results

#### Validation of the priming tasks administered before the tDCS experiment

**Figure 2:**
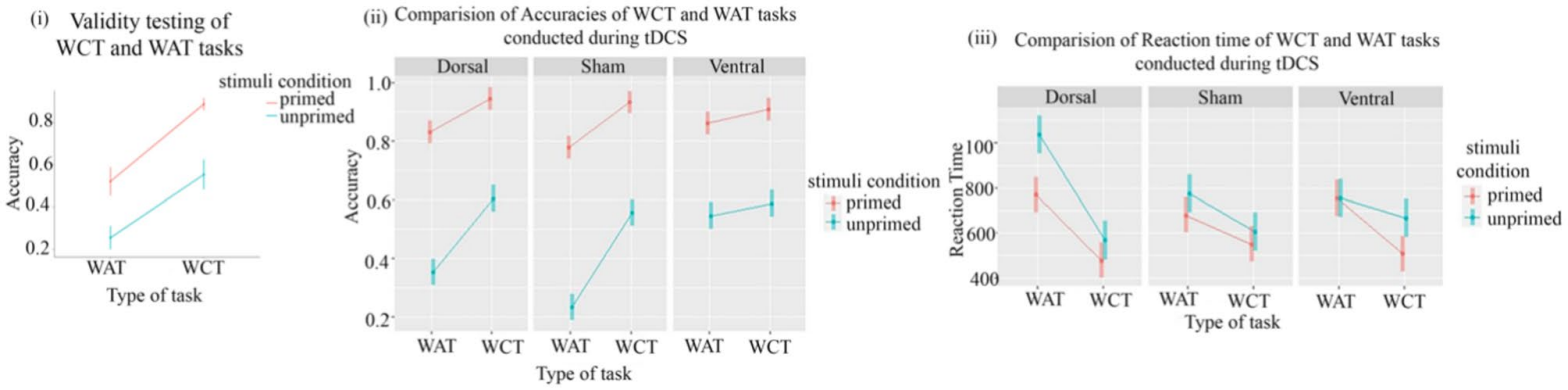
The results of the LMEMs used in the study with- (**i**) LMEM 2 showing the priming effect in WCT to be larger than in WAT in the behavioural assessment conducted before the tDCS experiment; (**ii**) During the tDCS experiment, LMEM 3 shows the priming effect to be larger for WCT compared to sham similar to LMEM 2. It also shows the accuracies improve in both primed and unprimed conditions of WAT following ventral stimulation compared to sham; (**iii**) LMEM 4 & 5 shows that the reaction time in primed and unprimed WAT conditions gets delayed following dorsal stimulation (compared to sham). Interestingly, the ventral stimulation exhibits no such effects.

LMEM 1a investigated the effect of task-type (WAT and WCT), priming-condition (Primed/Unprimed), and task-list on the ACC. There was no significant effect of task-list variation (β = 0.3, df= 51, F = 2.02, p = 0.1), indicating that the three-word lists (lists 1, 2, and 3) were equivalent. Similar results were obtained for RTs in LMEM 1b. LMEM 2a (see Fig. 2i) -which excluded task-list variation, identified significant main effects of task-type (std.β = 1.97, SE = 0.09, df = 44.41, 95% CI [− 1.89, 5.83], p < 0.001), priming-condition (std.β = − 1.15, SE = 0.09, df = 112.22, 95% CI [1.11, − 3.41], p < 0.001), and an interaction of task-type and Priming-Condition (std.β = − 1.15, SE = 0.13, df =1058.74, 95% CI [0.66, − 2.04], p < 0.001).

The LMEM 2a and b revealed a significant priming effect for both WAT (diff = 1.15, SE = 0.09,  z = 12.83, p < 0.001) and WCT (diff = 1.84, SE = 0.1, z = 18.51, p < 0.001). This can be seen in Fig. 2i, where the priming effect for ACC was larger in the WCT task (compared to WAT). The WCT accuracies were at the ceiling in prime condition, with no room for additional tDCS-induced improvement. For RTs (LMEM 2b, not shown in the figure), the main effects of task-type (std.β = 0.03, SE = 0.01, 95% CI [0.04, − 0.02], p < 0.001) and priming-condition (std. β = − 1.20, SE = 0.03, 95% CI [− 1.23, − 1.17], p < 0.001) were found to be significant. No interaction effect of Task-Type and Priming-Condition was found. This indicated that the responses were faster for WCT compared to WAT in primed compared to unprimed conditions for both tasks.

The power analysis showed a 100% power at alpha = 0.05 to detect the observed differences in both LMEM models 2a [95% CI (96.38, 100)] and 2b [95% CI (69.15, 100)].

#### Behavioural analysis of the priming tasks administered during tDCS stimulation

The LMEM 3 evaluated the effect of stimulation session, task type, and priming condition on ACCs of both tasks. The effect of ventral tDCS (std.β = − 2.09e^−02^, SE = 6.9e^−03^, df = 8.9e^+03^, t = − 3.021, p = 0.003) significantly differed from sham, whereas dorsal stimulation (std.β = 1.22e^−02^, SE = 6.9e^-03^, df = 8.9e^+03^, t = 1.76, p = 0.07) show a trend level significant. The main effects of task-type (std.β = − 5.24^e−02^, SE = 4.88e^−03^, df = 8.9e^+03^, t = − 10.726, p = 2e^−16^) and priming-condition (std. β = − 3.97e^−01^, SE = 8.5e^−03^, df = 8.9e^+03^, p = 2e^−16^) were significant. Two significant interaction effects are: (A) task-type and stimulation-session (std. β = − 2.52e^−02^, SE = 6.9e^−03^, df = 8.9e^+03^, t = − 3.647, p = 0.0003); and (B) priming-condition and stimulation-session (std. β = − 6.44e^−02^, SE = 1.2e^−02^, df = 8.9e^+03^, t = − 5.355, p = 8.77e^−08^).

A pairwise comparison of estimation of marginal means showed that the prime condition (0.87 ± 0.016) had significantly (β = 0.396, p < 0.001) higher ACCs than the un-primed condition (0.48 ± 0.017) irrespective of task-yype and stimulation-session. The means and standard deviations of primed and unprimed conditions for both the tasks following the three stimulation sessions are shown in Table 2 and Fig. 2(ii). It was observed that in the sham Condition, the priming effect was larger (t = 100.21, p < 0.001) for the WAT (0.54 ± 0.003) than for the WCT (0.377 ± 0.003). This was also found in LMEM 2 (Fig. 2i) in the validation of the task that was carried out before the tDCS stimulation experiment. Similarly, during dorsal stimulation, the WAT priming effect (0.48 ± 0.0034) was larger (t = 86.07, p < 0.001) than the WCT priming effect (0.34 ± 0.0034). However, in the ventral stimulation, the WAT priming effect (0.316 ± 0.0034) and the WCT priming effect (0.32 ± 0.0038) did not differ (t =  − 1.23, p = 0.6). These results show that the magnitude of difference observed between the priming effects of two tasks (WCT > WAT) compared to the sham condition remains intact following dorsal stimulation. However, this difference is negligible (i.e., PE^WCT^ ≅ PE^WAT^) in ventral conditions (Fig. 2ii). The negligible difference could be due to enhanced accuracies (t = 19.44, p < 0.01) for WAT following ventral stimulation (1.405 ± 0.043) compared to dorsal (1.18 ± 0.043). A similar enhancement in the accuracy (t = 34.47, p < 0.01) compared to sham (1.01 ± 0.043) was also observed. These differences were mainly driven by enhanced accuracies in unprimed conditions (0.543 ± 0.023) (see Table 2). Such a statistically significant difference was not seen for WCT.

**Table 2:** Mean and standard deviation of accuracies and reaction times for both the priming conditions (primed and unprimed condition) following dorsal, ventral and sham stimulation.

Stimulation-Session	WAT-primed	WAT-unprimed	WCT-primed	WCT-unprimed
	Mean ± std	Mean ± std	Mean ± std	Mean ± std
Accuracies (ACC)				
Sham	0.778 ± 0.0198	0.234 ± 0.0232	0.933 ± 0.0198	0.556 ± 0.0233
Ventral	0.862 ± 0.019	0.543 ± 0.023	0.945 ± 0.019	0.587 ± 0.023
Dorsal	0.833 ± 0.019	0.353 ± 0.023	0.945 ± 0.019	0.605 ± 0.023
Reaction times (RT)				
Sham	680 ± 40.5	776 ± 43.2	550 ± 40.5	607 ± 43.3
Ventral	755 ± 40.5	555 ± 43.2	507 ± 40.4	667 ± 43.5
Dorsal	770 ± 40	1037 ± 43.2	477 ± 40.5	567 ± 43.2

The LMEM 4 estimated the effect of stimulation session (dorsal, ventral, and sham), task type (WAT vs WCT), and priming condition (primed/unprimed) on RTs of each trial. Similar to LMEM 3, the main effects of task-type (std. β = 111.656, SE = 6.2, df = 8901, t = 18.005, p = 2e^−16^) and priming-condition (std. β = 111.525, SE = 10.8, df = 8901, t = 10.32, p = 2e^−16^) were significant. The interaction effect of task-type and stimulation session was significant for both ventral (std.β =  − 46.836, SE = 8.77, df = 8901, t = -5.34, p = 9.55e^−08^) and dorsal stimulation (std. β = 34.367, SE = 10.8, df = 8901, t = 10.32, p = 9.18e^−05^) contrasted with sham. Similarly, the interaction effect for priming and stimulation sessions was significant for both dorsal stimulation (std.β = 67.246, SE = 15.23, df = 8901, t = 4.41, p = 1.05e^−05^) and ventral stimulation (std. β =  − 35.570, SE = 15.27, df = 8901, t =  − 2.33, p = 0.01).

Estimation of marginal means shows (Table 2 and Fig. 2iii) that the response times in sham stimulation were faster for the prime condition than the unprimed condition for both WAT and WCT (similar to LMEM 2b). Contrary to our expectation for WAT, RTs for both primed (770 ± 40.5) and unprimed conditions (1037 ± 43.2) were significantly slower following dorsal stimulation compared to the reaction times of sham stimulation (primed : 680 ± 40.5, and unprimed :776 ± 43.2). Such slowing down is not seen for primed (477 ± 40.5) and unprimed (567 ± 43.2) conditions in the WCT when compared to sham (primed :550 ± 40.5, unprimed :607 ± 43.3). No difference is seen in RTs following ventral stimulation for WAT or WCT.

Taken together, tDCS-induced modulation (stimulation vs sham) of behaviour was seen mainly for WAT, whereas WCT showed no tDCS-modulation of performance. Specifically, LMEM 3 and 4 indicate that accuracy is enhanced for WAT following ventral stimulation, but RTs are delayed following dorsal stimulation compared to sham. The power analysis showed a 100% power at alpha = 0.05 to detect the observed differences in both LMEM models 3 [95% CI (69.15, 100)] and 4 [95% CI (96.38, 100)].

### Priming effect in the ERPs

**Figure 3:**
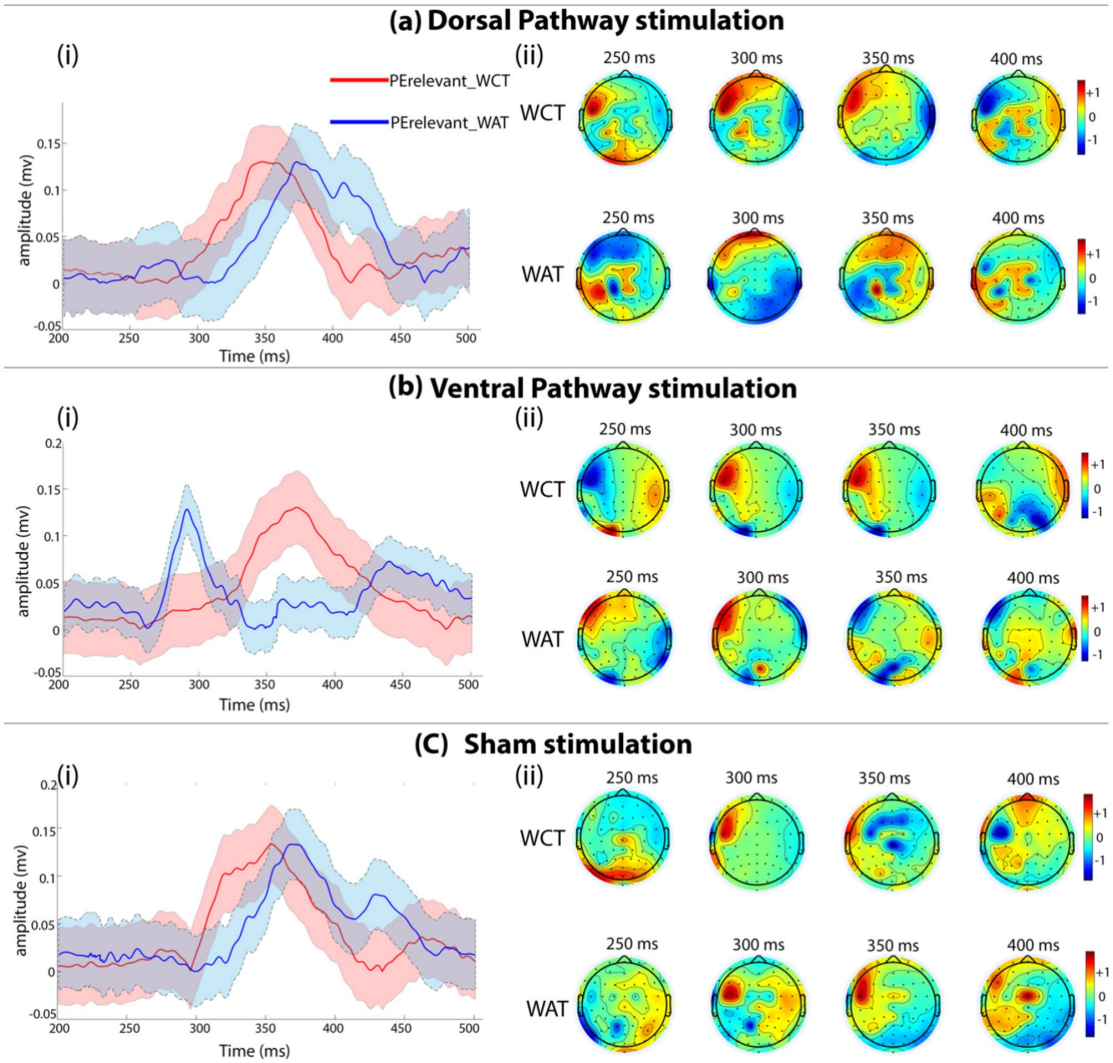
(**a**)(i), (**b**)(i), and (**c**)(i) show the temporal evolution of the priming effect for dorsal, ventral and sham stimulation. (**a**)(ii), (**b**)(ii), and (**c**)(ii) show spatial topographies and evolution of relevant priming effects for dorsal, ventral, and sham stimulation. It can be seen clearly that latency was affected in ventral stimulation, though significant effects (p < 0.05) in amplitude for dorsal stimulation were also reported.

The priming effects in the behavioural variables (ACC and RT) provided an understanding of the facilitation of the underlying neural processes due to tDCS. Subsequently, it became important to analyze the neural correlates from the ERPs. Figure 3 shows the temporal and spatial evolution of the relevant priming effects (PE^Relevant_WAT^ and PE^Relevant_WCT^) for (a) dorsal, (b) ventral, and (c) sham stimulation-session. As expected for language processing and word priming^28^, the spatial topographies were majorly concentrated in left frontal, temporal, and frontotemporal electrodes. It was interesting to see that latency changes only in ventral stimulation. In ventral stimulation (Fig. 3b(i)), the priming effect around 280 ms has decreased latency and sharp peak for WAT compared to the priming effect in dorsal and sham stimulation. Similarly, the spatial topography of the priming effect of WAT in ventral stimulation shows an early positive peak at around 280 ms and a negative peak at ~ 350 ms. The spatial topography of the effect corresponds to the areas in the left frontal and temporal regions (Fig. 3b(ii)). Such early activation and sharp peaking are not seen in the waveforms for dorsal (Fig. 3a(i)) and sham (Fig. 3c(i)) stimulation, nor are they observed for WCT for ventral stimulation (Fig. 3b(i)).

**Figure 4:**
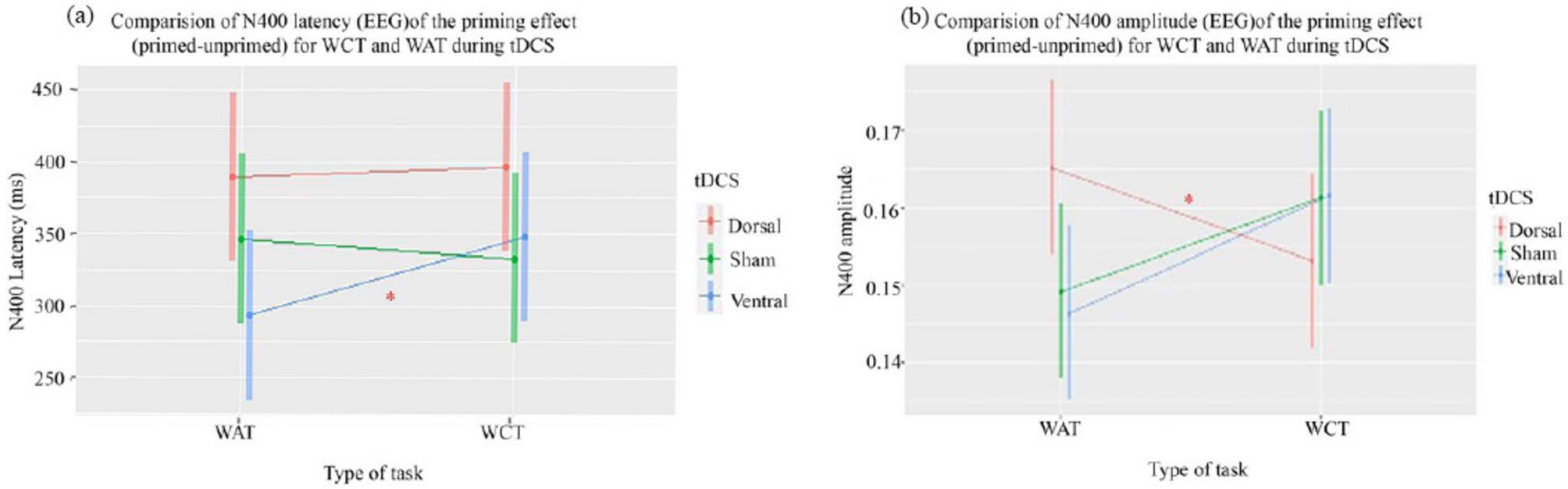
The results of LMEM 5 and 6 on priming effects (**a**) LMEM 5 shows N400 latency of the priming effect (primed—unprimed) to decrease in ventral stimulation for WAT only; and (**b**) Similarly, LMEM 6 shows the amplitude of N400 in the priming effect (primed-unprimed) to increase in dorsal stimulation for WAT only.

The LMEM 5 (Fig. 4a) investigated the effect of stimulation session (dorsal, ventral, and sham) and task type (WAT and WCT) on N400 latency obtained from PE^Relevant_WAT^ and PE^Relevant_WCT^. Only the main effect of the ventral condition compared to the sham was found significant (std. β = 8.3750, SE = 3.38, df = 95, t = 2.47, p < 0.01). Estimation of marginal means revealed latency in ventral stimulation (344.1 ± 4.27) to be significantly lesser (t = 14.50, p < 0.05) than sham (358.6 ± 4.27). Similarly, The LMEM 6 (Fig. 4b) investigated the effect of stimulation session (dorsal, ventral, and sham) and task type (WAT and WCT) on N400 amplitude obtained from PE^Relevant_WAT^ and PE^Relevant_WCT^. It was found that the amplitude during dorsal stimulation was significantly different from sham stimulation (std. β = 0.011, SE = 0.003, df = 95, t = 2.72, p < 0.01). The interaction effect of Task-type (WAT vs WCT) and stimulation-session (dorsal vs sham) was also significant (std. β = 0.008, SE = 0.003, df = 95, t = 2.64, p < 0.01). Estimation of marginal means shows that amplitude for WAT (0.165 ± 0.005) is significantly higher (t = 18.11, p < 0.001) than WCT (0.153 ± 0.005) for dorsal stimulation. On the other hand, the amplitude (mean ± std) of WAT for ventral (0.146 ± 0.005) and sham (0.149 ± 0.005) was significantly lower than WCT (ventral (0.162 ± 0.005), sham (0.161 ± 0.005)). Taken together with the output from LMEM 5 and 6, it can be said that WAT leads to a decrease in the latency of priming effects following ventral tDCS and an increase in the amplitude of the priming effect following dorsal stimulation. Interestingly, no significant stimulation-related changes in N400 latency or amplitude could be seen for WCT. The power analysis demonstrated a power of 82% [95% CI (71.75, 91.52)] in LMEM 5 and 84% [95% CI (73.79, 93.36)] in LMEM 6 at an alpha level of 0.05 to detect the observed differences.

## Discussion

The present study examined the alignment of behaviour with selective stimulation of reading pathways using tDCS to understand the pattern that maximizes the response. To this, we selectively stimulated the dorsal and ventral reading networks and recorded the ERPs while subjects performed the word priming tasks. The impact of the degree of involvement in a task on the tDCS effect was examined by two types of tasks, WCT and WAT, each with varying cognitive demands. Similarly, the effect of specific dorsal network stimulation (sub-lexical) on the tDCS effect was compared to ventral (lexical) network stimulation. The study found that tDCS improved participants' performance in a task (WAT) where there is room for improvement. Whereas WCT, which already reached the ceiling effect in performance and exhibited larger ERP amplitude during sham, failed to show any modulation (improvement) following tDCS. Only ventral stimulation enhanced accuracy in WAT, accompanied by decreased latency in the priming effect at N400. Similarly, dorsal stimulation slowed down the RTs in WAT associated with increased amplitude in the priming effect (primed—unprimed), contrary to the typical understanding that an increase in the signal's amplitude indicates improved performance^29^. One possible explanation could be that the dorsal stimulation facilitating the sub-lexical processing could be "maladaptive" and might interfere with the efficient functioning of the lexical route, which is needed for WAT. As a result, enhanced activation of neuronal resources with enhancement in amplitude might happen. Taken together, it can be said that tDCS works on a brain network with potential for improvement, and the performance can be maximized by appropriately stimulating the brain region underlying the behaviour.

The present study provides neurophysiological evidence that tDCS is more effective in less optimally utilized networks. The N400- an electrophysiological marker of processing of words in a semantic memory system, was found to vary in latency (for WAT with ventral stimulation) and amplitude (for WCT with dorsal stimulation). The N400's early contribution to psycholinguistic research highlighted the rapid detection of semantic changes, with the congruity effect appearing around 200–400 ms into critical word processing, regardless of its form^27^. Physiologically, N400 latency typically remains stable with changes in stimulus characteristics, a detail whose theoretical significance we are just beginning to comprehend^28^. Conversely, the reduced amplitude may suggest weakened post-synaptic potentials in the same neurons, activation of fewer neurons within a group, or decreased temporal synchronization among generating neurons^27,28^. The decrease in latency in the present study indicated that tDCS facilitated access to the semantic memory for the primed words. Similarly, a reduction in amplitude suggests that the retrieval from the semantic memory areas can be made at a lower pooling of task-related neurons. Altogether, the neural correlates of tDCS emphasize that when neuronal resources are maximally utilized during WCT (larger ERP amplitude) in the baseline condition, tDCS fails to enhance the neuronal response further. Whereas for WAT, which has relatively higher cognitive demand, there is sub-optimal utilization of neuronal resources (smaller ERP amplitude) and room for enhancement following tDCS. For such findings, Benwell et al. (2015) provided a working hypothesis, where they suggested that tDCS might facilitate the activation of task-relevant neurons to cross the optimal threshold where there is uncertainty in engaging the task-relevant neurons. Supporting this, multiple studies provided behavioural evidence that the tDCS effect is influenced by baseline performance^1,30^. Studies have also shown that an optimal level of task difficulty is needed^31,32^, and only individuals with poorer performance improve following tDCS compared to individuals with higher performance^33–35^. Kasahara et al. (2013) showed a similar result when performing two tasks (calculation and choice reaction tasks) in an fMRI scanner pre- and post-tDCS stimulation. They found that individuals with bilateral parietal activation failed to show tDCS-induced behavioural responses. In contrast, individuals with only left-dominant activation showed faster RTs, irrespective of the type of montage being administered. They suggested that the effectiveness of tDCS largely depends on the degree of endogenous neuronal activation.

The present study also found that the ventral (lexical) network enhanced performance in reading tasks that relied heavily on semantics. Whereas, worsening performance was seen during stimulation of the dorsal (sub-lexical) network. It is plausible that stimulation of the ventral network, which is naturally involved in processing semantic stimulus, establishes more robust connectivity within the underlying network. Previous study report that the increased coupling between the regions involved in a picture naming task (right temporoparietal and anterior cingulate cortex) for the repeated stimulus correlates strongly and persistently with greater priming magnitude across participants^11^. Our previous studies on the priming of famous faces showed that priming is not a purely local phenomenon but entails the interaction between the areas^36–38^. The network-interaction analysis revealed that the pathway for the flow of information (faces) from the fusiform gyrus to the prefrontal cortex via the middle temporal cortex is bypassed by priming, resulting in faster identification of the primed faces. We believe a similar approach to the present data can potentiate our claim and enhance our understanding regarding differential response associated with stimulation type.

On a similar line but from a different perspective, the brain engages more neuronal resources when the sub-lexical network is forcefully engaged following dorsal stimulation, which is not a usual processing network for tasks invoking semantic information. As a result, the stimulus is taken as unfamiliar when repeated and reflected by enhanced ERP amplitude. On the other hand, the brain naturally engages the lexical network to process the semantic task, causing interference with the output generated from the forceful engagement of the sub-lexical network. In such cases, extra time may be needed to resolve the conflict, causing a delay in the reaction time.

Overall, tDCS facilitates neuronal plasticity, but it depends on the performance levels and the network being stimulated and whether or not desired behavioural improvement is manifested in the output.

## Conclusion

The study investigated the effect of performance level and network specificity on tDCS effectiveness by examining behavioural and electrophysiological (EEG) correlations derived using a novel online tDCS-ERP approach. To this, dorsal and ventral reading networks were stimulated, and ERP was recorded while participants performed two reading tasks with differing degrees of performance. It was found that tDCS was more effective for the tasks in which performance was below the ceiling and where neuronal resources facilitating the behaviour were sub-maximally utilized. The effects on ERP latency and amplitude suggested that stimulating the network associated with the behaviour facilitates underlying neuronal processing, whereas stimulation of a network unrelated to the task may result in a maladaptive response. The uniqueness of the present study is that it includes a control task and controlled stimulation of a specific network to reveal that alignment of cognitive task with stimulation of specific cortical site underlying the behaviour is important to maximize the tDCS benefit.

## Data Availability

The datasets generated during the current study are not publicly available as prior permission was not obtained from the participants but are available from the corresponding author SB on reasonable request.
